# Epidemiology Investigation of stroke among Mongolian and Han population aged over 45 in Inner Mongolia

**DOI:** 10.1038/srep45710

**Published:** 2017-04-04

**Authors:** Chunyu Zhang, Tian Lan, Yan Zhe, Baolige Hu, Guohua Zhang, Juan He, Zhiguang Wang, Mingfang Jiang, Riletemuer Hu

**Affiliations:** 1 Department of Neurology, The Affiliated Hospital of Inner Mongolia Medical University, China

## Abstract

To discuss the status of epidemiology of stroke in the Mongolian and Han population aged over 45 years and to understand the treatment and prevention of stroke. Data collected on stroke populations aged over 45 years in the six areas in Inner Mongolia were analyzed by using stratified multi-stage cluster sampling. The prevalence rate of stroke in patients aged over 45 years in Inner Mongolia was 4.62%. The stroke prevalence rate increased with age in both males and females, the Han and Mongolian populations, and was higher in males than in females in Inner Mongolia. The prevalence rate of stroke in the Mongolian population was higher than in the Han population. The incidence rate of stroke in patients aged over 45 years in Inner Mongolia was 0.28%, of which the rate of relapsed ischemic stroke was 23.29%. The proportion of ischemic stroke in the stroke patients was higher than hemorrhagic stroke. The prevalence and incidence rates of stroke in patients aged over 45 years in Inner Mongolia were high. The prevalence rate of stroke in both the Han population and the Mongolian population increased with age. Ischemic stroke was the major form of stroke.

Stroke is a cerebral blood circulation disorder that can cause acute cerebral blood circulation obstacles for various reasons, such as atherostenosis, arterial occlusion, and arteriorrhexis in the brain. Stroke causes temporary or permanent nerve dysfunction, and has high prevalence, death, and disability rates^1^. From 1990 to 2010, the number of strokes worldwide doubled to 33 million. Currently, there are 16.9 million stroke patients each year worldwide, and the prevalence rate of stroke is 0.26%. The burden of stroke has increased worldwide because of the increasing sizes and aging of populations^2^,^3^, especially in low- and middle-income countries^4^,^5^. Economic development improved quality of life, and Western influence has affected disease patterns and aging. The occurrence of stroke in young patients greatly affects the economic situation and labor productivity in our country. The prevention and control of stroke is comprehensive in big cities and the developed eastern area of China because of good economic and medical condition. However, the epidemiological investigation of stroke in the western areas of China is less comprehensive because of bad economic and medical conditions. The prevention of stroke in minority areas apparently lags behind the developed provinces and cities, which does not benefit the health work in minority areas^6^,^7^.

Inner Mongolia is located along the northern border of China, which is an alpine region without convenient transportation. The diet is simple there, but the high intake of salt and fat may cause hypertension and cardiovascular disease. Hence, it is meaningful to raise the levels of the prevention of chronic diseases in these areas, as well as to improve the quality of life and health care and to construct a harmonious society by enhancing the study of the comprehensive prevention of stroke.

In this study, we investigated the status of the epidemiology of stroke in patients aged over 45 years among the Mongolian and Han populations in Inner Mongolia. The objective was to understand the treatment and prevention of stroke, which could provide a basis for making reasonable and effective prevention policies. Standardized management was used in the stroke populations. Establishing individual health records could lay a solid foundation for a prospective cohort study on stroke patients.

## Materials and Methods

### Sample population

We used stratified multi-stage cluster sampling to select six areas in Inner Mongolia-Hohhot, Baotou, Hulunbuir, Bayannaoer, Xilinguole League, and Xingan League-because of their unique geographical location. The six areas were chosen by using the primary cluster sampling, and then relevant county seats were randomly selected using the secondary cluster sampling. The county seats were divided into two levels (urban and rural) according to the economic conditions. A corresponding number of communities were selected according to the sample size, and 20% communities were selected as a reference. The number of samples was determined by the proportion of the population in the six areas. We selected 10,779 residents aged over 45 years, including the Mongolian population, the Han population, and other nationalities. The human related study was approved by the ethics committee of First Affiliated Hospital of Inner Mongolia Medical University and each patient provided informed consent.

### Diagnostic criteria

We used the standard formulated at the Fourth National Cerebrovascular Disease Conference^8^ to diagnose stroke, and confirmed it by imaging examination. To improve the accuracy of the investigation, three attending doctors reached a consensus of diagnosis when it was difficult to make a decision. If the attending doctors could not reach a consensus, then another senior doctor would participate in the discussion and make the decision. The leaked individuals in the first stage were checked. Each case was discussed and analyzed repeatedly according to the detailed medical history and physical examination. The head of the project made the final diagnosis combined with the examination results. Ischemic stroke was diagnosed by the criteria used in Trial of Org 10172 in Acute Stroke Treatment (TOAST) classification^9^.

### Investigation method

Questionnaires were used for this cross-sectional study. The preparations and the field survey were conducted from Jan 2013 to Dec 2014. Follow-ups, second reviews, second interviews, analyses, and data analyses were conducted from Jan 2015 to Dec 2015. The investigation was divided into two stages. In the first stage, agreement was obtained from the family members of households for interviewers from the Center for Disease Prevention and Control to collect basic information about screening and individual symptoms. In the second stage, trained neurologists re-checked the confirmed and suspected stroke cases mainly using data collected from the concentrated survey and the telephone survey. They carefully examined the medical history of physical and neurological examinations with reference to the hospitalization records and laboratory data. They checked the results according to the diagnostic criteria of stroke.

### Analysis of risks

The NIH Stroke Scale (NIHSS) is used for stroke severity measure in this study. Fifteen categories of brain function that are commonly affected by stroke are measured, and each item is scored between 0 and 4 points, including: best gaze level (0–2 points), consciousness level (0–3 points), consciousness questions level (0–2 points), consciousness commands level (0–2 points), left motor arm (0–4 points), right motor arm (0–4 points), left motor leg (0–4 points), right motor leg (0–4 points), limb ataxia (0–2 points), sensory (0–2 points), visual level (0–3 points), facial palsy level (0–3 points), best language (0–3 points), dysarthria (0–2 points), extinction and inattention (0–2 points). Chi-square test was used to assess the difference of scores in each risk factor.

### Statistical analysis

EpiData 3.1 was used to input the data and SPSS 13.0 was used for the data analysis. The enumeration data were analyzed by the χ^2^ test, and the results were according to mean number ± standard deviation. The statistical significance of differences between males and females, Han population and the Mongolian population were evaluated using the four-fold table chi-square test; the differences in the of the education levels, careers, family structures were analyzed by rows × columns chi-square test; and the differences in different age periods were analyzed using linear trend chi-square test^11^,^11^. P < 0.05 was considered statistically significance.

### Ethic Statement

The human related study was approved by the ethics committee of First Affiliated Hospital of Inner Mongolia Medical University and each patient provided informed consent.

## Results

### Characteristics of the sample population

This survey obtained complete data for 10,779 people aged over 45 years from six areas of Inner Mongolia. Five hundred and fifty-eight patients suspected of stroke were primary-screened, and the response rate was 99.28%. Four patients suspected of stroke did not reply, because they were not available, could not be contacted, or refused to be investigated. Four hundred and ninety-eight patients were diagnosed with stroke. In the Han population, there were higher proportions of stroke in female patients, patients with a minimum education level of junior middle school, and patients living with a spouse. There were significant differences between the education levels in the Han and Mongolian populations, as well as careers and family structures (Tables 1 and 2).

### Statistical results

The prevalence rate of stroke in patients aged over 45 years in Inner Mongolia was 4.62%, and the standardized prevalence rate was 4.64%. The standardized prevalence rates of stroke in males and females were 2.56% and 2.07%, respectively (Table 3). The standardized prevalence rates in the Han and Mongolian populations were 3.95% and 0.02%, respectively. The prevalence rate of stroke was higher in males than in females aged over 45 years (x^2^ = 4.854^a^, P = 0.028), and there was a significant difference between them. The prevalence rate of stroke in the Mongolian population was higher than in the Han population (x^2^ = 12.808^b^, P = 0.002), and there was significant difference between them (Table 4).

The prevalence rates of stroke in patients aged over 45 years increased with age, and there were significant differences between age groups (x^2^ = 253.301, P = 0.000) (Table 5). The prevalence rates of stroke in patients aged over 45 years were higher in males than in females, in which the prevalence rates of stroke in patients aged between 45 and 55 years were higher in females than in males; while the prevalence rates of stroke in patients aged between 55 to 65 years, 65 to 75 years and over 75 years were higher in males than in females. The prevalence rate of stroke patients increased with age, and there were significant differences between them (male: χ^2^ = 132.086, p = 0.000; female: χ^2^ = 121.725, p = 0.000) (Table 5).

The prevalence rates of stroke in the Mongolian and Han populations increased with age, and there were significant differences between age groups (χ^2^ = 245.474, P = 0.000) (Table 6). The prevalence rates in the Mongolian population from 45 to 55, 55 to 65, 65 to 75 years and over 75 years were significantly higher than in the Han population, especially in the 55 to 65 age group, in which the prevalence rate was twice as high as in the Han population. Overall, the prevalence rate of stroke patients aged over 45 years was higher in the Mongolian population than in the Han population. There were significant differences between the age groups (Han population: χ^2^ = 209.609, P = 0.000; Mongolian population: χ^2^ = 48.459, P = 0.000) (Table 6).

### The incidence and recurrence of stroke

Thirty cases of stroke were diagnosed from 01 January 2014 to 31 December 2014, including two death cases. The incidence rate of stroke was 0.28%. Four hundred and ninety-eight stroke patients were surveyed, among which stroke did not reoccur in 382 patients; the number of stroke patients who relapsed once, twice, three times, and four times were 73 (14.66%), 33 (6.63%), 6 (1.2%) and 4 (0.8%), respectively (Table 7). The relapsed patients accounted for 23.29% of all stroke patients. Cerebral infarction recurred in 30 cases, and lacunar infarction recurred in two cases. Cerebral hemorrhage recurred in one case, and subarachnoid hemorrhage recurred in one case.

### Classification of stroke

Among the stroke patients, there were 435 cases of cerebral ischemic stroke (87.35%) and 63 cases of hemorrhagic stroke (12.65%). There were eight cases of cerebral embolism among the cerebral ischemic stroke patients (1.84%) and three cases of subarachnoid hemorrhage among the hemorrhagic stroke patients (4.76%) (Table 8). In the Han population, the proportion of cerebral infarction in stroke patients was higher than cerebral hemorrhage in stroke patients. There were significant differences between the Mongolian and Han populations in the classification of stroke (χ^2^ = 6.511^a^, P = 0.011). The proportions of large-artery athero-sclerosis (LAA) and small-artery occlusion lacunar (SAO) were higher in the TOAST classification of cerebral ischemic stroke. There were no significant differences between the Mongolian population and the Han population in the TOAST classification of cerebral ischemic stroke (χ^2^ = 3.497^a^, P = 0.478).

### Analysis of risk factors in stroke

A case-control study on the risk factors of stroke was conducted in 1: 4 matched stroke patients of Mongolian and Han population by age, sex and origin. We found there were significant differences of scores in risk factors between Mongolian and Han populations. In Mongolian population, there were significant differences in the scores of hypertension, diabetes and drinking; while in Han population, there were significant differences in the scores of hypertension, diabetes and hyperlipidemia. Hence, hypertension and diabetes were the common risk factors in Mongolian and Han populations (Tables 9 and 10).

## Discussion

The results showed significant differences between nationalities in the prevalence of stroke. In 1985, a national epidemiology investigation of stroke^12^ showed that the standardized prevalence rate of cerebrovascular disease was 0.31%. In 2003, Feigin *et al*.^13^ reviewed the existing studies of the prevalence rate of stroke in 10 countries. The standardized prevalence rate of stroke in people aged over 65 years ranged between 4.6% and 7.3%. According to the reports from different countries worldwide, the prevalence rate of stroke ranged from 0.4% to 1%. The prevalence rate of the white (except Hispanic) in the United States, the black (except Hispanic), Hispanics, and people in Pacific region were 2.3%, 4%, 2.6%, and 1.6%, respectively.

Our country had a high incidence rate of stroke. The distribution of stoke was consistent with hypertension. The prevalence rate of stroke in the northern region was higher than in the southern region. The median age of onset in the eastern region was 61 years, 59 years in the central region and 57 years in the western region^14^, which might be related to economics and medical conditions. Three large-scale surveys on stroke in China were conducted in 1985, 1986, and 1991^9^,^15^,^16^. Because the survey year and age–sex structure of the investigation area varied in these previous studies, their results might differ from those in our study.

In Inner Mongolia, the prevalence rate of stroke was lower than in developed countries. However, because of the backward economic and medical conditions in Inner Mongolia, it was still higher than in other regions in China. Because the population aged over 75 years was lower than other age groups, the prevalence rate was significantly lower than other age groups. According to the demographic yearbook^17^, the population aged over 70 years in the Mongolian population was lower than other age groups; therefore, the prevalence rate of patients aged over 75 years was significantly lower than in the Han population. The prevalence rates of patients aged 45 to 55, 55 to 65, and 65 to 75 years were significantly higher than in the Han population, which might be related to geographical location, culture, education, and alcohol consumption in Inner Mongolia. The prevalence rates of stroke in the age groups studied in the Mongolian population were significantly higher than in the Han population. Similarly, the prevalence rate of hypertension in Mongolian population was significantly higher than in the Han population^18^. Hypertension and stroke in the Mongolian population might be related to the genes ACEDD, CYPCT, and TT^19^. The proportions of herdsman and peasants were very high in this survey. The number of patients with a junior middle school education level was higher than the number of patients with other education levels. The proportion of total annual family income less than RMB 30,000 was high. The awareness and understanding of stroke and the incidence of physical examinations in the middle-aged and high-risk population were worse than in developed areas. The risk factors of hypertension, diabetes, and fat combined with low income could increase the incidence of the disease and increase the burden on low-income countries and areas^20^.

The number of stroke patients worldwide will increase by 90,000 each year in the next few years because of the increased number of people aged over 65 years. In addition, the prevalence rate of stroke and its different subtypes increase with age^19^,^20^. Fang XH reported^21^ that the standardized prevalence rates of stroke in patients aged from 55 to 59, 60 to 64, and over 65 years were 9.5%, 11.7%, and 14%, respectively. The genetic differences between sex and income had an obvious relationship with the occurrence of stroke^22^,^23^. The present study found that the prevalence of stroke in males was higher than in females, which might be related to estrogen, which was a protective factor for cerebrovascular disease. Estrogen could regulate lipid metabolism, thus inhibit intimal hyperplasia caused by platelet aggregation, stress, and mechanical damage. The prevalence rate of stroke in males aged 55 to 65 years was twice as high as in females and accelerated significantly, and in males aged over 65 years was higher than in females, which might be related to the unhealthy living habits, such as heavy smoking and alcohol consumption.

Feigin *et al*.^24^ reviewed the prevalence rates in the reports of stroke in high-income, middle-income, and low-income countries. From 1970 to 2008, the prevalence rate in high-income countries decreased by 42%. The prevalence rate, especially in people aged 75 years in middle-income and low-income countries, increased by 100%. In addition, from 2000 to 2008, the incidence rate was higher than in high-income countries for the first time. The prevalence rate of stroke in China ranged from 0.12% to 0.22%, while it was 0.28% in Inner Mongolia, which might be related to the following reasons: the investigated population aged over 45 years; the investigated areas were mainly rural and pastoral; economic and medical conditions were relatively backward. The proportion of recurrence of stroke in patients was high, which might be related to the limited knowledge about the incidence and recurrence of stroke. LAA was the most common, followed by SAO. This result was in accordance with previous reports^25^. The proportions of LAA and SAO were high in China, and cardiogenic cerebral embolism was very common in Western countries. The proportion of symptomatic atherosclerotic intracranial arterial stenosis in Asians was higher than in the white^26^. The TOAST classification could help in the prognosis, diagnosis, and prevention of ischemic stroke. In China, hypertension is closely related to LAA^27^. The proportions of LAA and SAO were high in this study, which indicates that key in the prevention and treatment of stroke is preventing and treating the risk factors of hypertension and diabetes. The proportion of ischemic stroke was high in both the Mongolian population and the Han population. The incidence rate of ischemic stroke increases by 8.7% every year in China^28^. Hu RL reported^29^ that the knowledge of hypertension and the adherence to a medication regime were higher in the Han population than in the Mongolian population. In this study, we found there were significant differences in risk factors in stroke between Mongolian and Han populations. Hypertension and diabetes were the common risk factors in Mongolian and Han populations. Therefore, it is important to strengthen the education about the risk factors of stroke for patients, high-risk populations, community hospital workers, and primary hospital workers.

## Conclusion

The prevalence rates, incidence rates, and recurrence rates of stroke patients aged over 45 years were higher in the Mongolian population than in the Han population. The proportion of ischemic stroke was also high. Hence, we should take effective and reasonable measures according to characteristics of epidemiology in local populations.

## Additional Information

**How to cite this article:** Zhang, C. *et al*. Epidemiology Investigation of stroke among Mongolian and Han population aged over 45 in Inner Mongolia. *Sci. Rep.* **7**, 45710; doi: 10.1038/srep45710 (2017).

**Publisher's note:** Springer Nature remains neutral with regard to jurisdictional claims in published maps and institutional affiliations.

## Figures and Tables

**Table 1:** Different situation number of stroke.

Situations	General data (complete)	Suspected of stroke	Response data	Diagnosed with stroke
Number	10779	558	554	498

**Table 2:** Basic characteristics of the sample population.

	Han population	Mongolian population	Total	χ^2^	P value
Education level					
Illiterate	419(3.89%)	94(0.87%)	513	126.732^a^	0.000
Primary school	1931(18.23%)	296(2.80%)	2227		
Junior middle school	3373(31.85%)	838(7.91%)	4211		
Senior high school, technical secondary school, junior college	2365(22.33%)	635(5.81%)	3000		
Bachelor or above	433(4.09%)	206(1.94%)	639		
Career					
Herdsman	686(6.48%)	155(1.46%)	841	93.101^a^	0.000
Peasant	1180(11.14%)	285(2.68%)	1465		
Worker	1216(11.48%)	243(2.29%)	1459		
Science, education, health	523(4.94%)	127(1.20%)	650		
Administration, police	540(5.10%)	221(2.09%)	761		
Service industry, individual	907(8.56%)	266(2.51%)	1173		
Housework	1030(9.73%)	300(2.83%)	1330		
Retirement	1916(18.09%)	395(3.73%)	2311		
Other careers	523(4.94%)	77(0.73%)	600		
Family structure					
Living with spouse	4944(46.69%)	835(7.88%)	5779	246.825^a^	0.000
Living with spouse and children	2611(24.66%)	960(9.07%)	3571		
Living with children	435(4.11%)	165(1.56%)	600		
living alone	439(4.15%)	83(0.78%)	522		
Others	92(0.87%)	26(0.25%)	118		

^a^Indicated using rows × columns chi-square test; p < 0.05, showed there were significant difference.

**Table 3:** The prevalence rate and standardized prevalence rate in male and female, Mongolian and Han population.

Characteristics	Total number	Number of patients	Prevalence rate (%)	Population proportion	Standardized prevalence rate (%)
Sex					4.64
Male	5238	266	5.08	0.5049	2.56
Female	5541	232	4.19	0.4951	2.07
Nationality					4.39
Han population	8521	362	4.25	0.9297	3.95
Mongolian population	2069	124	5.99	0.0032	0.02
Others	189	12	6.35	0.0671	0.43

Standardized by constituent ratio of male and female, Mongolian and Han population aged over 45 according to data of national census 2000.

**Table 4:** Comparison between male and female, Mongolian and Han population.

Characteristics	Stroke		Total number	χ^2^	P value	OR(95% CI)
	Yes	No				
Sex						
male	266(5.1%)	4972	5238	4.854^a^	0.028	1.224(1.022, 1.466)
female	232(4.2%)	5309	5541			
Nationality						
Han population	362(4.2%)	8159	8521	12.808^b^	0.002	
Mongolian population**	124(6.0%)	1945	2069			
others**	12(6.3%)	177	189			

^a^Indicated using four-fold table chi-square test; ^b^indicated using rows × columns chi-square test; level of significance with *p < 0.05 compare with Han population; level of significance with *p < 0.01 compare with Han population.

**Table 5:** The prevalence rate in male and female of different ages.

Age	Male			Female			Total		
	Total number	Number of patients	Prevalence rate(%)	Total number	Number of patients	Prevalence rate(%)	Total number	Number of patients	Prevalence rate(%)
45–55	2202	24	1.09	2193	25	1.14	4395	49	1.11
55–65	1599	98	6.13	1790	71	3.97	3389	169	4.99
65–75	920	95	10.33	1063	93	8.75	1983	188	9.48
≥75	517	49	9.48	495	43	8.69	1012	92	9.09
Total	5238	266	5.08	5541	232	4.20	10799	498	4.61
χ^2^			132.086			121.725			253.301
P value			0.000			0.000			0.000

Using linear trend chi-square test; level of significance with p < 0.01, the prevalence rate of stroke increased with ages.

**Table 6:** The prevalence rate in Mongolian and Han population of different ages.

Age	Han population			Mongolian population			Total		
	Total number	Number of patients	Prevalence rate(%)	Total number	Number of patients	Prevalence rate(%)	Total number	Number of patients	Prevalence rate(%)
45–55	3302	31	0.94	999	17	1.70	4301	48	1.11
55–65	2719	110	4.05	610	54	8.85	3329	164	4.93
65–75	1649	142	8.61	314	42	13.38	1963	184	9.37
≥75	851	79	9.28	146	11	7.53	997	90	9.03
Total	8521	362	4.25	2069	124	5.99	10590	486	4.59
χ^2^			209.609			48.459			245.474
P value			0.000			0.000			0.000

Using linear trend chi-square test; level of significance with p < 0.01, the prevalence rate of stroke increased with ages.

**Table 7:** The recurrence of stroke.

Characteristics	Non recurrence	Relapse 1 time	Relapse 2 times	Relapse 3 times	Relapse 4 times
Relapse	382(76.7%)	73(14.66%)	33(6.63%)	6(1.20%)	4(0.80%)

**Table 8:** Classification of stroke.

Classification	Cases	Percentage
Cerebral ischemic stroke	435	87.35%
cerebral embolism	8	1.84%
Hemorrhagic stroke	63	12.65%
subarachnoid hemorrhage	3	4.76%

**Table 9:** Analysis of risk factors in stroke of Mongolian populations.

Risk factors	Stroke		Total	χ^2^	P value	OR(95% CI)
	Yes	No				
Hypertension						
Yes	99(79.84%)	156	255	96.259^a^	0.000	8.656(5.368, 13.959)
No	25(20.16%)	341	366			
Diabetes						
Yes	1814.52%	39	57	5.260^a^	0.022	1.990(1.095, 3.615)
No	106(85.48%)	457	563			
Hyperlipidemia						
Yes	23(18.55%)	103	126	0.301^a^	0.586	0.869(0.526, 1.436)
No	101(81.45%)	393	494			
Coronary heart disease						
Yes	11(8.87%)	65	76	1.653^a^	0.199	0.645(0.330, 1.264)
No	113(90.32%)	431	544			
Smoking						
Yes	39(31.45%)	130	169	1.375^a^	0.241	1.292(0.841, 1.983)
No	85(68.55%)	336	421			
Drinking						
Yes	35(28.22%)	81	116	9.229^a^	0.002	2.015(1.275, 3.185)
No	89(71.78%)	415	504			
Family history						
Yes	15(12.10%)	40	55	1.995^a^	0.158	1.569(0.836, 2.943)
No	109(87.90%)	456	565			

^a^Indicated using chi-square test.

**Table 10:** Analysis of risk factors in stroke of Han populations.

Risk factors	Stroke		Total	χ^2^	P value	OR(95%CI)
	Yes	No				
Hypertension						
Yes	237(65.47%)	756	993	20.561^a^	0.000	1.735(1.365, 2.206)
No	125(34.53%)	692	817			
Diabetes						
Yes	77(21.27%)	175	252	20.387^a^	0.000	1.965(1.460, 2.646)
No	285(78.73%)	1273	1558			
Hyperlipidemia						
Yes	95(26.24%)	305	400	4.513^a^	0.034	1.333(1.022, 1.740)
No	267(73.76%)	1143	1410			
Coronary heart disease						
Yes	58(16.02%)	179	237	3.410^a^	0.065	1.353(0.981, 1.865)
No	304(83.98%)	1269	1573			
Atrial fibrillation						
Yes	9(2.49%)	56	65	1.596	0.207	0.634(0.310, 1.294)
No	353(97.51%)	1392	1745			
Smoking						
Yes	137(37.85%)	496	633	1.642^a^	0.200	1.169(0.921, 1.483)
No	225(62.15%)	952	1177			
Drinking						
Yes	103(28.45%)	450	553	0.940^a^	0.332	0.882(0.684, 1.137)
No	259(71.55%)	998	1257			
Family history						
Yes	56(22.93%)	162	218	5.012^a^	0.025	1.453(1.046, 2.018)
No	306(77.07%)	1286	1592			

^a^Indicated using chi-square test.
